# Evidence of Pneumopericardium after Elective Ovariectomy in a Peritoneopericardial Diaphragmatic Hernia-Affected Dog: A Case Report

**DOI:** 10.3390/ani14040633

**Published:** 2024-02-16

**Authors:** Debora Campanile, Mariateresa Cafaro, Serena Paci, Michele Panarese, Giammarino Sparapano, Marina Masi, Antonio De Simone

**Affiliations:** 1 Ospedale Veterinario Atheryon, via Grecia, 2, 76125 Trani, Italy; 2 Clinica Veterinaria Polispecialistica Associata, via Parco del Lauro, 72, 70044 Polignano a Mare, Italy

**Keywords:** pneumopericardium, peritoneopericardial diaphragmatic hernia, congenital malformation, cardiac tamponade, dog, peritoneopericardial diaphragmatic repair

## Abstract

**Simple Summary:**

Congenital malformations in dogs are still poorly understood and, often, inadequately treated. This report describes the clinical signs, the diagnosis and the treatment of a peritoneopericardial diaphragmatic hernia, an abnormal communication between the abdominal and thoracic cavities, in a dog after its accidental diagnosis due to the development of an intraoperative complication that could have led to progressive cardiorespiratory changes and, potentially, to death.

**Abstract:**

Peritoneopericardial diaphragmatic hernia (PPDH) is an opening between the pericardial sac and the pleuroperitoneal membrane. Pneumopericardium is an infrequent complication of PPDH. This condition is a serious circumstance in which free gas accumulates in the pericardial sac. The present report describes the occurrence of pneumopericardium and pericardial effusion after elective ovariectomy in a dog affected by PPDH. The presence of an umbilical and diaphragmatic hernia was highlighted during ovariectomy, and a pneumopericardium was seen during an X-ray exam. At the time of admission to the hospital, the dog was asymptomatic. The diagnosis was performed by X-ray and ultrasonographic exams. Computed tomography examination confirmed the diagnosis and directed for a surgical approach of the congenital defect. Surgery resulted in resolution of PPDH and of pneumopericardium.

## 1. Introduction

Peritoneopericardial diaphragmatic hernia (PPDH) is a congenital anomaly observed in dogs and cats, and it appears to be a common incidental finding. Its origin lies in a dysembryogenesis, resulting in an opening or communication between the pericardial sac and the pleuroperitoneal membrane [1,2].

Organs that are normally located within the peritoneal cavity may be displaced into the pericardial sac [3]. There are three theories regarding the embryogenesis of PPDH in dogs and cats: (1) failure of the lateral pleuroperitoneal folds and the ventromedial pars sternalis to unite during division of the coelom into abdominal and thoracic cavities; (2) faulty development of the dorsolateral septum transversum or rupture of a thin tissue membrane in this area allowing peritoneal and pericardial communication; (3) prenatal injury to the septum transversum or to the fusion site of the of the septum transversum and the pleuroperitoneal folds [4]. Although the development abnormality responsible for the malformation has not been identified, PPDH is likely caused by failure of formation or fusion of the septum transversum in the sterno-costal triangle structure [5]. In humans, it can be caused by either trauma or congenital anomalies [6]. In cats and dogs, PPDH is due solely to congenital anomalies because there is no connection between the diaphragm and pericardium in healthy animals, and the PPDH could not be the result of trauma [7]. PPDH with a large defect exists too, where parts or whole organs displace into the thoracic cavity, or PPDH with a small diaphragm defect. A suspected breed predisposition exists for Weimaraner dogs and domestic longhair cats [4]. Theoretically, all visceral organs may be displaced, but the liver, gallbladder, and small intestine are the most herniated. Physical examination may reveal muffled heart sounds, decreased lung sounds, thoracic borborygmi, or an empty abdomen on palpation [8].

PPDH is usually diagnosed with thoracic radiographs but, in some cases, positive contrast radiographic exam, ultrasonography, echocardiography and computed tomography (CT) may be useful [5]. Affected animals are asymptomatic or show nonspecific clinical signs such as acute vomiting, labored respiration, exercise intolerance, collapse, fever, and diarrhea [7]. The diagnosis of PPDH is often an incidental finding. For asymptomatic patients, careful monitoring is recommended, but a surgical approach is not the norm. Surgery is recommended in symptomatic patients and consists in closing the congenital defect [9]. Intraoperative complications ranging from easily manageable events to potentially fatal outcomes such as hypoventilation, hypotension, cardiac arrhythmias, hemorrhage, pneumothorax, and hypoxia. In the post operative period, there may be complications such as pericardial effusion, pericardial cysts, cough, pneumopericardium, pneumothorax, vomiting, regurgitation, hyperthermia, wound infection, or incisional dehiscence [3]. Pneumopericardium is an infrequent complication of PPDH, and it is rarely reported in small animals [8]. The causes of pneumopericardium in dogs and cats include thoracic trauma, positive pressure ventilation, pulmonary–pericardial communications, tracheal rupture, laparoscopic procedure, alveolar rupture associated with cough and bronchospasm, and infectious causes [10].

The present report describes the occurrence of pneumopericardium and pericardial effusion in a PPDH-affected dog that underwent elective ovariectomy. 

## 2. Materials and Methods

### 2.1. Case Description

**Figure 1:**
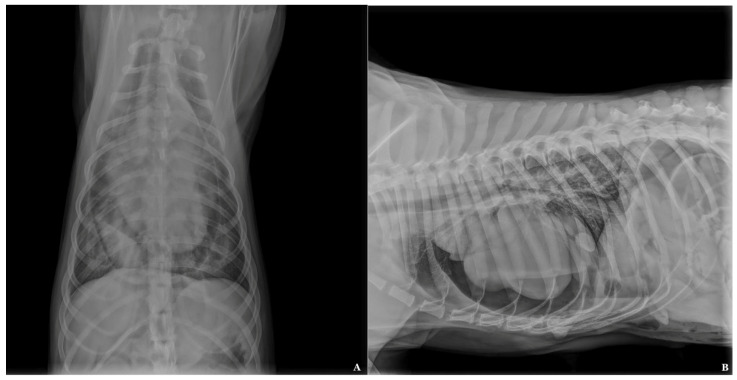
(**A**) Dorsoventral and (**B**) lateral radiographic projection of the thorax showing a pneumopericardium with severe gas collection outlining cardiac silhouette and pericardial thin border.

An 18-month-old, 34 kg, spayed female, mixed breed dog was referred to Atheryon Veterinary Hospital (AVH) for treatment of pneumopericardium. A few hours earlier the dog had been neutered at the clinic of the referring veterinarian, who had highlighted the presence of umbilical and diaphragmatic hernias during surgery. Immediately after surgery, on a lateral and dorsoventral radiographic projection of the thorax, a pneumopericardium was seen with severe gas collection outlining the cardiac silhouette and pericardial thin (Figure 1A,B). At the time of presentation at the AVH, the owners did not report any clinical symptoms in anamnesis, but they reported that, although it was a young dog, it seemed lethargic and showed exercise intolerance. During the first clinical evaluation at AVH, the dog was bright and alert. Rectal temperature was 38.6 °C, heart and respiratory rates were within reference limits. Femoral pulses were strong and regular. Results of CBC, serum biochemical profile, and urinalysis were unremarkable. 

### 2.2. Diagnostic Imaging 

**Figure 2:**
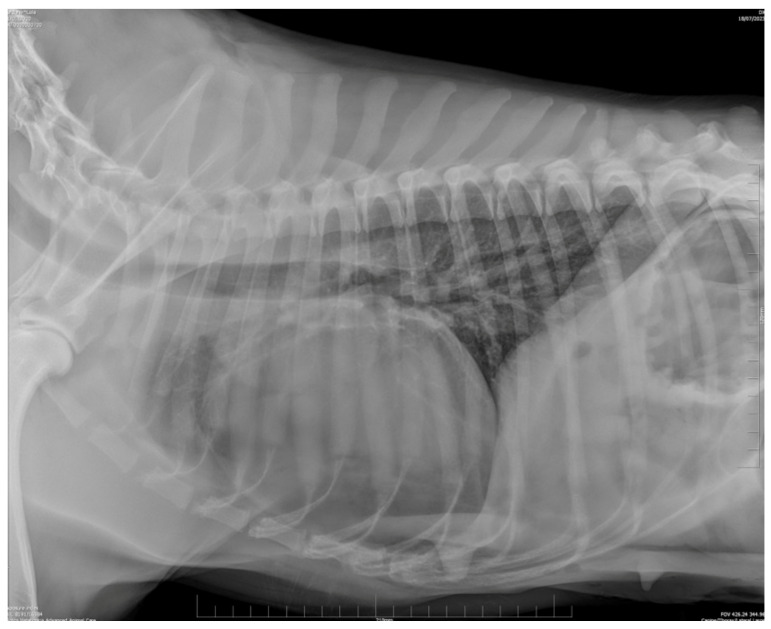
Latero-lateral (LL) thoracic radiography. Cardiomegaly due to pericardial effusion without cardiac tamponade was suspected and it was confirmed by the normal shape and diameter of lobar vascular structures. A thin gas layer is slightly visible in the cranial part of the pericardium showing time based partial resolution of gas collection.

Left latero-lateral (LL) thoracic radiography showed severe cardiomegaly and dorsal tracheal displacement. Within the radiopaque pericardial border, a minimal amount of gas opacity was observed cranially. The shape and thickness of the cranial lobar pulmonary vasculature were consistent. Time-based partial resolution of the pneumopericardium and pericardial effusion without cardiac tamponade were suspected (Figure 2). 

**Figure 3:**
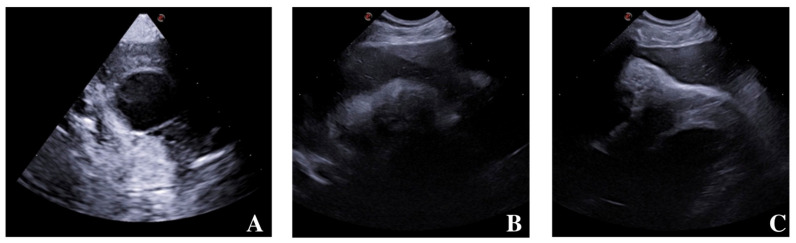
Ultrasonography. (**A**) Gallbladder displacement beside heart chambers, fluid- and gas-filled pericardium. (**B**) Heart chambers and part of a sharply marginated liver lobe within pericardial sac. (**C**) Anechoic fluid effusion around heart chambers and multiple reverberating spot suggesting resolving/partial pneumopericardium.

An ultrasonographic cardiac exam was performed in right lateral recumbency, using standard long and short axis views with both microconvex and sectorial probes, showing anechoic fluid surrounding the heart in the pericardial sac, combined with multiple thin hyperechoic reverberant spots, whose position changed with recumbency. The cardiac chambers were normal in size and shape, with normally filling and uncollapsed right atrium, and the dog was slightly tachycardic during the examination. In the caudal portion of the pericardial sac, a sharply marginated liver lobe was seen close to the cardiac apex, together with a rounded/oval shaped fluid-filled structure matching with a partially displaced gallbladder. A peritoneal-pericardial diaphragmatic hernia and mixed fluid–gas effusion in the pericardium were strongly suspected, and a CT scan was requested, without needing a preventive pericardiocentesis (Figure 3A–C).

Computed tomography examination and the subsequent hernia repair was undertaken during the same anesthesia. The dog was premedicated with 5 mcg/kg IV of dexmedetomidine (Sedadex, Dechra, Northwich, UK) and 0.2 mg/kg IV of methadone (Semfortan, Dechra). Meloxicam (0.2 mg/kg SC, Metacam, Boehringer Ingelheim/Rhein Germany) and cefazoline (30 mg/kg IV) were administered prior to surgery.

General anesthesia was induced using a mixture of ketamine (1 mg/K, Lobotor, ACME, Malvern, PA, USA) and propofol (1 mg/kg, Proposure, Abbott, Chicago, IL, USA) through slow intravenous injection. An 11 mm cuffed endotracheal tube was connected to a rebreathing circuit. The dog received isoflurane (Isoflo, Esteve, Barcelona, Spain) in a gas mixture of 40% oxygen and air. The patient was mechanically ventilated by use of a respirator operated in a volume-controlled mode with tidal volume of 15 mL/kg, an inspiratory to expiratory ratio of 1:2, a 4 cm H_2_O PEEP, and a Paw limit of 20 cm H_2_O. The respiratory rate was adjusted to maintain an ETCO2 of 35–45 mmHg.

**Figure 4:**
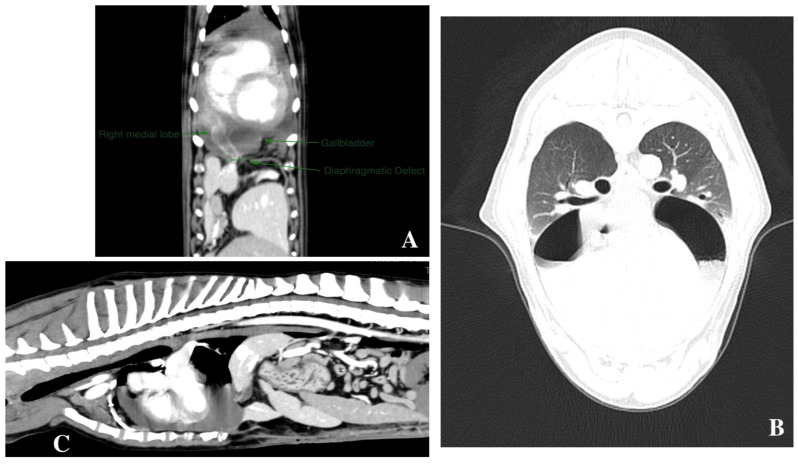
Computed tomography (CT) examination. (**A**) Dorsal multiplanar reconstruction (MPR) showing the diaphragmatic defect and both liver and gallbladder displacement in the pericardial sac. (**B**) Pneumopericardium, lung window transverse scan showing gas–fluid collection layers within the pericardial sac. (**C**) Sagittal MPR reconstruction showing topography of the postoperative pericardial effusion (PPE) and the pneumopericardium.

Immediately after induction, the dog received NaCl solution (5 mL/kg/h IV), and a constant-rate infusion of ketamine 0.5 mg/kg/h (Lobotor, ACME) and dexmedetomidine 1 mcg/kg/h (Sedadex, Dechra) in the same syringe pump was started. A continuous lead II electrocardiogram (ECG); heart rate; systolic, diastolic, and mean arterial pressure (determined at the right dorsal radial artery by use of a non-invasive oscillometric technique); oxygen saturation, as measured by pulse oximetry; FiO_2_; end-tidal isoflurane concentration; ETCO2; Paw; plateau airway pressure; tidal volume; and minute ventilation were continuously monitored throughout anesthesia. A 16-slice helical MDCT scanner was used (Siemens Somatom Emotion 16; Siemens Medical Engineer, Erlangen, Germany). The dog was placed in sternal recumbency, and a total body CT scan was acquired before and after 600 mgI/kg endovenous iodine-based contrast medium administration (Ultravist 300 mgI/mL; Bayer^®^, Leverkusen, Germany). CT scan was obtained with a craniocaudal direction, 1.5 mm slice thickness, 0.9 pitch, 110 kV and 130 mAs, 0.6 s/rotation. Soft tissue, bone, and lung windows were reconstructed from raw data using soft tissue and bone kernel, respectively, and 3D multiplanar reconstruction was obtained with a dedicated software (Horos, Pureview, Chelsea, MA, USA). The CT scan showed an enlarged pericardial sac containing both gas (in the dorsal portion) and fluid (ventrally) of about 30 HU density measured by ROI, surrounding the heart; in the caudal portion a vascularized, swollen, slightly hypodense, triangle-shaped part of the right medial lobe of the liver was seen on the right side just caudal to the right ventricle. The gallbladder was also displaced behind the cardiac apex in the pericardial sac (cystic duct was in place), together with part of the falciform ligament and fat. A diaphragmatic central/slightly right-sided ventral defect communicating with the pericardial sac was seen, measuring about 2 cm in diameter. Accessory findings were: slightly enlarged sternal lymphnodes and thymus, and, therefore, an anomaly of seventh sternebra and xiphoid process which were both half-splitted, with the latter interrupted by a large cartilage portion. A peritoneal-pericardial diaphragmatic hernia and pericardial fluid effusion and pneumopericardium, partial liver and gallbladder displacement were diagnosed. Congestion/ischemia of the hypoattenuating liver lobe was postulated (Figure 4A–C). 

### 2.3. Surgical Technique

**Figure 5:**
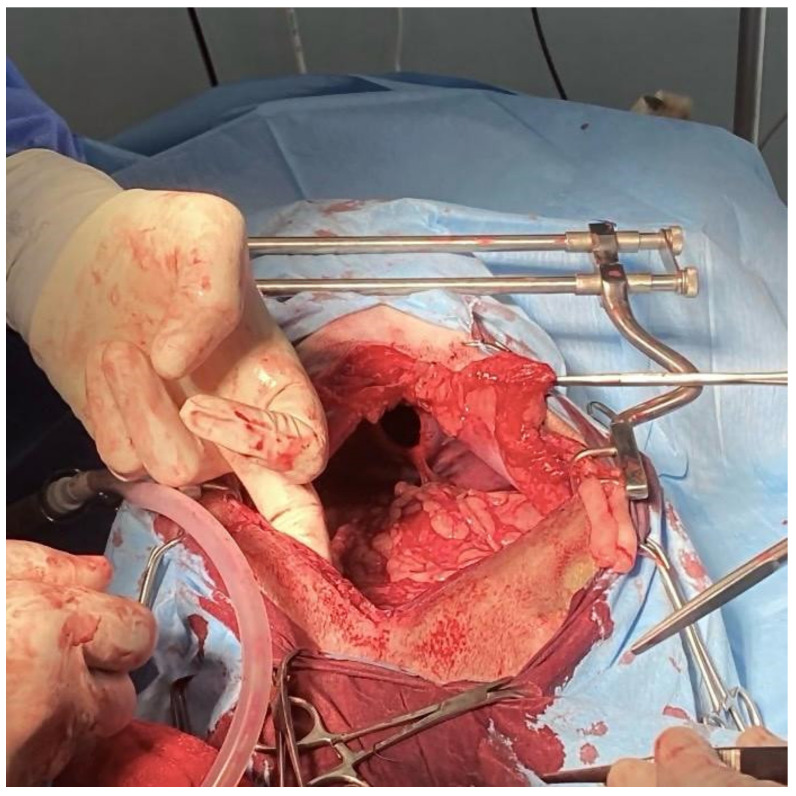
Peritoneopericardial hernia surgery visualization through a 4–5 cm opening in the ventral third of the diaphragm.

In order to proceed with the surgery, the dog was placed in dorsal recumbency for preparation of the surgical site, which involved clipping and cleaning with a 2% chlorhexidine scrub solution followed by application of isopropyl alcohol solution. Midline celiotomy and resection of the falciform ligament was performed to expose the hernia. The gallbladder and the quadrate liver lobe were found to be displaced into the pericardial sac through a 4–5 cm opening in the ventral third of the diaphragm (Figure 5). The gallbladder and the liver lobe were repositioned manually. No adhesion was observed, and the pericardial effusion was drained. The edges of the PPDH defect were opposed and closed using a simple continuous suture pattern with absorbable suture (PDS 2/0, Ethicon). Pericardiocentesis was made until achieving negative pressure. Upon closure, a unilateral 5 Fr thoracic drain was placed to enable post-surgical air and fluid removal if needed. The abdominal cavity was closed using a simple continuous suture pattern with absorbable suture for muscle (Vicryl 0, Ethicon, Cincinnati, OH, USA) and subcutaneous tissue (Monsyn 2/0, B Braun, Melsungen, Germany). The skin was closed with an intradermal absorbable suture (Monsyn 2/0, B Braun). The patient did not experience any complications during anesthesia.

### 2.4. Postoperative Management and Outcome

During hospitalization, the dog was treated with intravenous maintenance fluids (2 mL/kg/h; NaCl 0.9%, Braun). Postoperative analgesia was provided with a constant rate infusion of lidocaine (2 mg/kg/h, Lidor, Richter Pharma) IV, preceded by a loading bolus of 2 mg/kg IV and ketamine 0.1 mg/Kg/h IV (Lobotor, ACME) in the same syringe pump for 24 h after surgery. Meloxicam 0.1 mg/kg SC q 24 h (Metacam, Boehringer Ingelheim), 0.2 mg/kg q 4 h IV of methadone (Semfortan, Dechra), and cefazoline (30 mg/kg IV q 8 h) were administered during recovery. The patient recovered quickly and started eating again. Since the thorax drain was not productive anymore (no production of fluid or air) and the breathing pattern and the arterial blood gasses were both good, the thorax drain was removed on day 2 post-surgery. The following day, a follow-up echocardiographic examination was performed. No pericardial effusion or other cardiac abnormalities were found.

The dog was discharged 36 h after surgery without any complications; meloxicam (0.1 mg/kg PO q 24 h) was prescribed for pain relief for three consecutive days. A follow-up examination 1 month after hospital discharge was conducted, and the dog was clinically stable and in a general good condition. Furthermore, the owners reported a notable improvement in their dog’s vitality and greater propensity for physical activity and movement.

## 3. Discussion

Pneumopericardium is a serious condition in which free gas accumulates in the pericardial sac. In people, the most common cause of pneumopericardium is alveolar rupture. Other causes include blunt chest trauma, pneumonia, asthma, and neoplasia. Conditions such as acquired immune deficiency syndrome, ingestion of cocaine, and pyogenic lung abscesses may be associated with pneumopericardium [11]. Pneumopericardium is rarely reported in small animals and may be caused by spontaneous, traumatic, or iatrogenic causes. The documented causes of pneumopericardium in dogs and cats include thoracic trauma, positive pressure ventilation, pulmonary–pericardial communication, tracheal rupture, alveolar rupture associated with cough and bronchospasm, secondary to pneumonia, and lung abscess [10].

The key diagnostic features of pneumopericardium in thoracic radiographs is the presence of radiolucent air that completely outlines the epicardium all over the cardiac silhouette, including both auricles and the origin of the large central vessels [12].

PPDH is a congenital anomaly observed in dogs and cats. Animals with PPDH often have other concurrent congenital abnormalities such as midline defects (e.g., umbilical hernia, cleft palate, sternal abnormalities) and cardiac defects (e.g., ventricular septal defects, subaortic stenosis, pulmonic stenosis, atrial septic defect) [13]. PPDH is commonly seen as a congenital abnormality in several animals of the same litter. Therefore, breeding of affected animals is not recommended, and screening of littermates for PPDH should be performed [14]. Pneumopericardium has been described as a potential complication of PPDH [7]. In the present case report, the pneumopericardium was generated during ovariectomy by the passage of air from the previously surgically exposed abdomen, through a congenital defect of the diaphragm, thereby connecting the peritoneal and pericardial cavities. Iatrogenic induction of pneumopericardium by introduction of room air into the pericardial sac was used before the echocardiography as a tool to visualize the pericardial and cardiac masses [15]. The diagnostic pneumopericardiography induced hemodynamic effects like those due from cardiac tamponade [16]. Acute accumulation of air in the pericardial space may potentially cause serious hemodynamic changes such as acute hypotension, dyspnea, weakness, cardiogenic shock, and death [17]. In the veterinary literature, one case of pneumopericardium as a sequel of laparoscopic procedure associated with abdominal pressure of 10 mmHg has been documented [18]. In that case, following insufflation of the abdomen, signs of cardiac tamponade developed, resulting in death. Our dog recovered uneventfully from ovariectomy, and pneumopericardium was diagnosed after surgery, probably because slower filling of the pericardial cavity with air would likely allow time for pericardial distension and prevent severe signs of tamponade [19]. The evidence of pericardial effusion was probably due to the injury to the pericardial mesothelial cells and consequent bleeding and immune response; therefore, it is suspected to be also a consequence of migration of abdominal inflammatory fluid produced normally after surgery. These conditions are described as “post cardiac injury syndromes” and represent different clinical conditions characterized by an initial cardiac injury involving the pericardium, myocardium, and/or pleura and the subsequent inflammatory syndrome ranging from simple pericarditis to more complicated pleuropericarditis, cardiac tamponade, or pleural effusion [20]. The severity of clinical signs of pneumopericardium depends on the cause as well as the amount of the accumulated gas that may interfere with cardiopulmonary function. The dog described in the present report did not have clinical signs relating to PPDH, and the diagnosis was made incidentally after ovariectomy. A concurrent umbilical hernia, corrected during the first surgery, is a common associated finding with PPDH, with a remote possibility of umbilical remnants herniation; sternal anomalies are also frequently described [13]. Evidence regarding the surgical treatment and outcome of canine PPDH has been described in only a few reports. Treatment includes surgical herniorrhaphy or medical conservative treatment, particularly in the absence of clinical signs or in the presence of medical conditions limiting surgical repair. Based on scientific evidence, surgical treatment of PPDH had a high rate of success and low operative mortality [4,5,7]. Post-operative complications have been reported in 11 to 48% of dogs and include pneumothorax, cardiac arrhythmias, pneumopericardium, pneumothorax, pericardial steatitis, gastrointestinal signs, and obstructive portal hypertension [7,8]. Although surgical treatment did not show high rates of complications, dogs live apparently normal lives for many years without surgery. Further studies are needed to determine which dogs with PPDH would benefit from surgical versus conservative treatment [4,5,7]. In our case, although clinical signs associated with PPDH and pneumopericardium were absent in the dog, surgery was selected because computed tomographic abnormalities did not suggest that the lesion would be self-limiting. Therefore, there was concern for vascular injury (congestion/partial ischemia) of the herniated liver lobe and gallbladder. Surgery resulted in resolution of the PPDH and of the pneumopericardium.

## 4. Conclusions

The described case report represents a rare but relevant example of PPDH in a dog. Pneumopericardium and pericardial effusion are two consequences that can arise from this disorder, which is defined by improper communication between the pericardial sac and the pleuroperitoneal membrane. Diagnosis of PPDH can be challenging because of the non-specific presentation of this condition, and often, the finding of PPDH is incidental. An accurate diagnosis was made possible using several imaging modalities, such as computed tomography (CT), ultrasonography, and X-rays, which allowed for prompt surgical intervention. The treatment strategy of PPDH may be either surgical or conservative. Both surgical and conservative treatments provide good long-term survival. Surgery is recommended in symptomatic patients. This clinical case highlights the importance of early diagnosis and surgical repair of PPDH to prevent potentially fatal complications.

## Data Availability

Data are contained within the article.
